# Cow’s milk allergy: From allergens to new forms of diagnosis, therapy and prevention^☆^

**DOI:** 10.1016/j.ymeth.2013.08.005

**Published:** 2014-03-01

**Authors:** Heidrun Hochwallner, Ulrike Schulmeister, Ines Swoboda, Susanne Spitzauer, Rudolf Valenta

**Affiliations:** a Division of Immunopathology, Department of Pathophysiology and Allergy Research, Medical University of Vienna, Austria; b Department of Medical and Chemical Laboratory Diagnostics, Medical University of Vienna, Austria

**Keywords:** Milk allergy, Recombinant allergens, Diagnosis, Therapy, Microarray

## Abstract

The first adverse reactions to cow’s milk were already described 2000 years ago. However, it was only 50 years ago that several groups started with the analysis of cow’s milk allergens. Meanwhile the spectrum of allergy eliciting proteins within cow’s milk is identified and several cow’s milk allergens have been characterized regarding their biochemical properties, fold and IgE binding epitopes. The diagnosis of cow’s milk allergy is diverse ranging from fast and cheap *in vitro* assays to elaborate *in vivo* assays. Considerable effort was spent to improve the diagnosis from an extract-based into a component resolved concept. There is still no suitable therapy available against cow’s milk allergy except avoidance. Therefore research needs to focus on the development of suitable and safe immunotherapies that do not elicit severe side effect.

## 1 Introduction

### 1.1 History of cow’s milk allergy

The introduction of cow’s milk (CM) into alimentation has a very long tradition. It is reported that animal milk was included into the human diet approximately 9000 years ago. The domestication of cattle provided meat and milk as important components of our diet [1]. The production of cheese started with the ancient Greeks and Romans [2]. At the same time people in Northern Europe lost their lactose intolerance. Therefore lactase activity, a genetic trait, and animal husbandry, a cultural trait, represent an example for gene-culture co-evolution [3]. The first adverse reactions to CM that were described by Hippocrates (prior to 370 B.C.) were skin and gastrointestinal symptoms after CM consumption [4]. Five hundred years later, the Greek medical researcher Galen of Pergamum mentioned a causal relationship between these symptoms and milk consumption [5]. At the beginning of the 20th century observations of adverse reactions to CM became more frequent. The first reports mentioning diarrhea, growth retardation as well as anaphylactic shock after milk consumption were mainly published in the German literature [6–9]. The Swedish clinician Wernstedt proposed the term “idiosyncrasy” for this phenomenon [10].

### 1.2 Prevalence of cow’s milk allergy

Today CM is among the first foods introduced into an infant’s diet and accordingly is one of the first and most common causes of food allergy in early childhood. In fact, the term “allergic March” describes the typical early appearance of food allergy which precedes the subsequent development of respiratory allergy in children [11]. The reported prevalence of cow’s milk allergy (CMA) varies dramatically between studies which may be attributable to different methods used for diagnosis or differences in the ages of the studied populations [12]. Furthermore, geographical factors may influence the rates for prevalence. In general, the frequencies of self-reported adverse reactions to CM are much higher than the medically confirmed diagnoses, not only in children but also in adults [13]. A meta-analysis of relevant original studies since 1990 by Rona et al. [14] showed a variation in self-reported prevalence of milk allergy between 1.2% and 17%, whereas the prevalence in studies using a double-blind placebo controlled food challenge or an open challenge varied between 0% and 3% and in studies based on skin prick testing (SPT) and IgE assessment frequencies were between 2% and 9%.

Nowadays, it is reported that 0.6–2.5% of preschoolers, 0.3% of older children and teens and less than 0.5% of adults suffer from CMA [13]. The prevalence of CMA is increasing which may be explained by a decrease in breast feeding and an increased feeding with cow’s milk-based formulas [15].

Several factors which may increase the risk for developing CMA are described such as genetic predisposition for allergy (i.e., atopy), early ingestion of small amounts of CM and also factors related to the intestinal microbiome [16].

Interestingly, the majority of CM allergic infants outgrow their CMA. One study reports that 45–50% grow out at 1 year, 60–75% at 2 years, and 85–90% at 3 years of age [17]. Although another study did not confirm exactly these numbers and the time course for the development of tolerance, it is reported that CMA resolved in 19% of the children by 4 years of age, in 42% by 8 years of age, in 64% by 12 years of age and in 79% by 16 years of age [18]. The mechanisms underlying the development of clinical tolerance are not fully understood. Several factors may be involved in the development of tolerance. They may include a decline of IgE antibodies due to avoidance, the development of blocking IgG antibodies due to regular intake of CM and/or the presence of IgE antibodies against mainly conformational epitopes and not against sequential epitopes [19–21]. One study has shown that reactions to less than 10 ml of milk during an oral food challenge and large wheal size reactions in SPT predict a higher risk for persistence [22]. Several other studies have confirmed that low levels of milk-specific IgE and small wheals during SPTs are good indicators for resolution [23–25]. A recent observational study considered the severity of atopic dermatitis (AD) for the natural course of milk allergy and came up with a web-based calculator for the prognosis of milk allergy for infants younger than 15 months that is based on milk-specific IgE levels, SPT wheal sizes and severity of AD [26].

### 1.3 Spectrum of symptoms and immune mechanisms in CMA

The clinical symptoms of CMA may be elicited by different mechanisms. The immediate and IgE-associated mechanisms are responsible for approximately 60% of cow’s milk-induced adverse reactions. They may affect one or more organs. Typical IgE-associated symptoms appear immediately or within 1–2 h after CM ingestion and affect the skin, the respiratory system, the gastrointestinal tract and/or appear as systemic anaphylactic reactions in severe cases [13,27]. The IgE-mediated reactions affecting the skin comprise urticaria, angioedema, pruritus, rashes and flushing. Atopic dermatitis is usually T cell-mediated but T cell activation may be enhanced by IgE-facilitated allergen presentation [28]. Respiratory symptoms that appear immediately after CM ingestion are rhinoconjunctivitis, wheezing, coughing, asthma exacerbation and laryngeal edema [2,13]. Acute gastrointestinal symptoms include oral itching, abdominal pain, nausea, vomiting and diarrhea. CM is the third most common food component after peanuts and tree nuts that causes anaphylactic reactions, accounting for 10–19% of all food-induced anaphylactic cases [27,29] with cardiovascular collapse, syncope or incontinence as the most severe characteristics [13]. Furthermore, food-dependent exercise-induced anaphylaxis is reported to appear in infants who outgrew their allergy or after an oral immunotherapy [30,31].

Several mechanisms leading to the initial sensitization to CM proteins are discussed.

One hypothesis is that sensitization may occur before birth: In this context it has been shown that small amounts of food proteins consumed by pregnant women can reach the foetus via the placenta [32]. In fact, it has been speculated that IgE may be already produced by foetuses in early pregnancy and can be detected in cord blood [33].

The other possibility is sensitization early after birth through intake of CM. However, it is still controversially discussed if the early contact with CM proteins leads to sensitization or to clinical tolerance to CM. There is still an ongoing debate if babies should be exclusively breast fed [34–36]. Interestingly, also sensitization to human milk has been reported [37]. Oral intake or other mechanisms of sensitization such as sensitization via the skin or by inhalation have also been considered [38].

Usually clinical reactions start very early in life, after breast-feeding is stopped and CM is introduced in the diet, whereas only in rare cases symptoms appear already during lactation [27,37,39].

Besides IgE-associated mechanisms also non-IgE-mediated mechanisms of cow’s milk hypersensitivity do occur but they are difficult to diagnose [40]. Non-IgE-associated symptoms are characterized by a delayed onset, around 2 h to several days after CM consumption. Patients suffering from this form of hypersensitivity lack circulating CM protein-specific IgE and they show negative results in skin prick tests [27,41–44]. It is estimated that around 0.5% of all infants suffer from non-IgE-mediated CMA [45] whereas it seems to be more common in adults. The clinical symptoms affect mainly the gastrointestinal system including enterocolitis, proctitis, proctocolitis, enteropathy, irritable bowel syndrome, eosinophilic esophagitis and constipation [13,27,46]. The role of milk allergens in gastroesophageal reflux (GER), infantile colic and constipation is still under debate and needs further investigation [27]. Delayed respiratory symptoms include pulmonary hemosiderosis, chronic cough, tachypnea, wheezing and rales. Sometimes atopic dermatitis appears as a chronic symptom after CM ingestion. Hypersensitivity to CM in infants may be associated with pulmonary hemosiderosis and present chronic symptoms such as recurrent fevers, weight loss and failure to thrive [2,27,47,48].

The precise mechanisms leading to non-IgE mediated forms of CMA are still under debate. In principle, several immunological mechanisms may be responsible for non-IgE-mediated reactions to CM proteins [49]. Symptoms may be caused by cow’s milk-specific T cell responses, antibody-mediated mechanisms may involve Type II or Type III hypersensitivity mechanisms such as ADCC (antibody-dependent cell-mediated cytotoxicity) or complement activation [49,50]. Furthermore, Th1/Th2 imbalances are assumed to have an impact [51]. While atopy patch testing which measures CM allergen-specific T cell responses may be useful to assess T cell-mediated reactions neither the measurement of CM allergen-specific IgG nor IgA are useful for diagnosis of non-IgE-associated CMA [52–54].

It is also possible that patients suffer from mixed manifestations, elicited through IgE- and non-IgE-mediated reactions. Both humoral and/or cell-mediated mechanisms may induce symptoms, including acute and chronic manifestations. The clinical manifestations may appear as atopic dermatitis and eosinophilic gastroenteropathies (esophagitis and gastroenteritis) [27].

### 1.4 The spectrum of cow’s milk allergens

Cow’s milk contains around 30–35 g of proteins per litre and includes more than 25 different proteins but only some of them are known to be allergenic. Table 1 provides a short summary of the known CM allergens and their characteristics. Through the acidification of raw skim milk to pH 4.6 at 20 °C two fractions can be obtained: the coagulum containing the casein proteins which accounts for 80% and the lactoserum (whey proteins) representing 20% of the total milk proteins [55–57]. The casein fraction (Bos d 8, *Bos domesticus*) consists of four proteins which account for different percentages of the whole fraction: αS1-casein (Bos d 9, 32%), αS2-casein (Bos d 10, 10%), β-casein (Bos d 11, 28%) and κ-casein (Bos d 12, 10%) with αS1-casein being the most important allergen of the casein fraction [58]. Allergens found in the whey fraction are α-lactalbumin (Bos d 4), β-lactoglobulin (Bos d 5), immunoglobulins (Bos d 7), bovine serum albumin (BSA, Bos d 6) and traces of lactoferrin (Bos d lactoferrin). α-lactalbumin and β-lactoglobulin are the most important allergens of the whey fraction, accounting for 5% and 10% of the total milk proteins [55,59]. There are only few reports describing allergies to minor whey proteins such as immunoglobulin, BSA or lactoferrin [60].

A major problem of CMA is the fact that the human IgE response to CM proteins is characterized by a great variability and that no single allergen or particular structure has been identified that accounts for a major part of allergenicity in milk. Sensitization to several proteins occurs in approximately 75% of patients with CMA, with a great variability of the IgE response in specificity and intensity. The most frequently recognized allergens seem to be those which are most abundant in CM, namely caseins, β-lactoglobulin and α-lactalbumin [21,61]. For the definition of the clinically most relevant allergens it will be necessary to conduct extensive IgE binding studies in large populations of clinically well-defined CM allergic patients and an assessment of the allergenic activity of the individual allergen components [21]. At present there is still a lot of controversy about the prevalence of IgE reactivity to certain CM proteins. One of the reasons for this might be that study groups were selected based on different criteria and often were very small [21,59,61–66].

#### 1.4.1 Allergens in the casein fraction

The four caseins (αS1-, αS2-, β- and κ-casein) form ordered aggregates also termed micelles [67]. These complexes bind essential minerals, such as calcium phosphate, that would otherwise precipitate and would not be easily ingested [68]. Casein micelles are spherical aggregates with diameters ranging between 100 and 300 nm [69]. The α- and β-caseins form the interior of the micelles while κ-casein is located on the surface. The four casein molecules have little primary structure homology but they are all phosphorylated proteins and share biophysical features such as heat resistance. So far it has not been possible to resolve the three-dimensional structure of the individual caseins due to their highly rheomorphic structure. CM allergic patients are normally sensitized to several of the different casein proteins. Furthermore, there are amino acid sequence homologies of up to 90% between caseins from different mammals, like goat and sheep, and accordingly extensive cross-reactivity but there are also reports of selective sensitizations to caseins from certain animals [70–72].

Allergenic features were investigated in detail for αS1-casein, the major CM allergen which was expressed in recombinant form in *Escherichia coli*. CD spectroscopy analysis showed a primarily β-fold structure that kept its structure even upon heating up to 55 °C. However, caseins are easily digested in the gut which is a rather unusual feature for important allergens [73].

Using synthetic peptides and recombinant fragments epitopes of the major and minor CM allergens can be mapped. In contrast to respiratory allergens, that contain mainly conformational epitopes, several linear epitopes have been identified for food allergens [58,74–76]. Several sequential epitopes are distributed along αS1-casein. However, experiments showed that mainly intact αS1-casein or larger IgE-reactive fragments thereof are responsible for the induction of allergic reactions [58]. αS2-casein, ß-casein and κ-casein were characterized regarding their epitope distribution but data on allergenic activity are rare [77–81].

#### 1.4.2 The whey allergens

β-Lactoglobulin is a major whey protein of most mammalian species but is not found in the milk of rodents and human. It is a small protein with a molecular weight of 18.3 kDa, consisting of 162 amino acids and it naturally occurs in a dimeric form. The structure determination by X-ray crystallization as well as by NMR techniques [82–87] revealed a globular shape that is built up by an 8-stranded, antiparallel β-barrel with a 3-turn α-helix on the outer surface and a ninth β-strand flanking the first strand [88]. The function of this milk protein is not yet known but it belongs to the lipocalins, a protein superfamily, that binds hydrophobic ligands like cholesterol and vitamin D_2_ [82,88]. There are two major isoforms of beta-lactoglobulin, the genetic variants A (BLGA) and B (BLGB), which differ in amino acids 64 and 118 (aspartic acid and valine in BLGA, glycine and alanine in BLGB). It has been shown that two disulphide bonds account for high stability against proteases and acidic hydrolysis [55]. The allergenic potential of this molecule has been attributed to its high stability and the fact that β-lactoglobulin is not present in human milk [55,89]. However, recent data indicate that α-lactalbumin is the more important whey allergen [21].

α-Lactalbumin is a small (14 kDa), acidic, Ca^2+^ binding, monomeric protein that is stabilized by four disulphide bridges. It acts as a regulatory component in the galactosyltransferase system that synthesizes lactose [90] and it may interact with lipid membranes [91], stearic acid and palmitic acid [92]. A multimeric form of α-lactalbumin can induce apoptosis in tumor but not in normal cells [93–95].

α-Lactalbumin has become one of the best described and characterized molecules in protein science because of its ability to convert into a molten globule state under acidic pH [90] and in the apo-state (calcium-depleted state) under elevated temperatures. This was first described by Dolgikh in 1981 [96] as a compact state with fluctuating tertiary structure.

Sequence analysis showed a high degree of homology between the α-lactalbumin amino acid sequences from cows to human subjects and rodents, ranging at approximately 75% identity [97]. Furthermore, α-lactalbumin shows high thermal stability and refolding capacity [97].

**Fig. 1:**
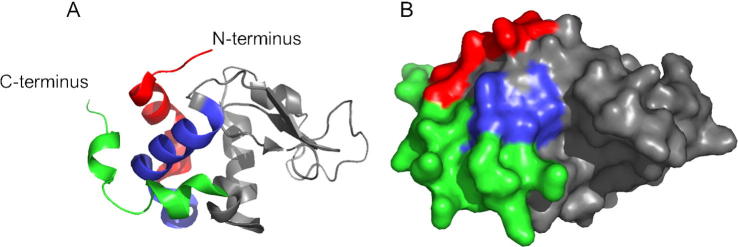
Ribbon (A) and molecular surface (B) presentations of α-lactalbumin. The N- and C-terminus are indicated in A. IgE-binding epitopes are colored and form a surface-exposed patch. The figure is taken from Hochwallner et al. [97].

Similar to the pollen allergens Aln g 4, Bet v 3, Bet v 4, Phl p 7, the fish allergen parvalbumin and the cockroach allergen Bla g 6, α-lactalbumin belongs to the family of calcium-binding proteins [98]. So far described calcium-binding allergens are members of the EF-hand calcium binding proteins, which are characterized by helix-loop-helix calcium-binding domains that consist of 12 amino acids. In contrast, α-lactalbumin has an “elbow-Ca^2+^-binding-loop”, which differs from the typical EF-hand motifs due to a more compact structure and the presence of only 10 amino acids in the domain. Two structural domains, a large α-helical domain at the N-terminus and a short β-sheet domain at the C-terminus are flanking the calcium-binding loop, which is made up of residues 79–88. On either side of the loop there are cysteine residues which form four disulfide bridges and in this way close the loop [99,100] and stabilize the structure of α-lactalbumin [90]. Using synthetic peptides it was possible to localize IgE epitopes within this allergen. They are mainly located at the N- and C-terminal end of the protein which appear in close vicinity on the surface of α-lactalbumin and define an IgE-reactive area on the protein shown in Fig. 1. The clustering of the IgE epitopes might play an important role for the efficient cross-linking of IgE antibodies on effector cells and might influence the allergenic activity of an allergen [97] as also described for other allergens such as Bet v 2, Phl p 5, Phl p 1, Phl p 2 and Bos d 5 which also seem to contain clusters of IgE epitopes [101–105].

Lactoferrin is an iron-binding glycoprotein with 703 amino acids and a size of 80 kDa that belongs to the transferrin family. Besides its function as a scavenger of free radicals and as an antioxidant, it has antimicrobial properties and plays an important role in defence against infections [57].

Bovine serum albumin has 582 amino acids, a weight of 66.3 kDa and contains 17 disulphide bonds [106]. BSA plays an important role in transport, metabolism, distribution of ligands, and osmotic pressure of blood and prevention of free radicals [107]. This allergen plays a role in CMA but also beef allergy [108].

## 2 Methods for diagnosis of cow’s milk allergy

A recently published article from the WAO describes the guidelines for the diagnosis of CMA in great detail [109]. Diagnosis of food allergy begins with a thorough clinical history, followed by diagnostic tests. To test if the patient suffers from IgE-mediated allergy it is possible to determine specific IgE antibodies in the serum using the CAP-FEIA System or UniCAP (Phadia, Uppsala, Sweden). High concentrations of food-specific IgE correlate with an increased risk of clinical symptoms [103,110]. Skin prick tests provide a fast method to detect sensitization [111]. But a positive test does not necessarily prove that the food is causal and does not unambiguously demonstrate an IgE-mediated allergy. It can only be confirmed by detection of allergen-specific IgE (e.g. false positive reaction in urticaria factitia patient). Atopy patch tests are used as diagnostic tools for non-IgE mediated reactions after cow’s milk consumption but there are no standardized reagents, methods of application and interpretation available [112]. The double-blind placebo-controlled oral food challenge (DBPCFC), the gold standard for the diagnosis of food allergies, and also for milk allergy, can only be performed after the suspected food is eliminated from the diet [113,114].

### 2.1 Double-blind placebo-controlled food challenge (DBPCFC)

The double-blind placebo-controlled food challenges are standardized tests and are performed to obtain a clear diagnosis and to prevent the patient from unnecessary diets [115]. However the number of allergy clinics performing DBPCFC routinely is still limited worldwide [116]. During these tests patients consume progressively increasing quantities of CM as described in a standardised protocol proposed by the European Academy of Allergy and Clinical Immunology in 2004 [117]. The challenge is stopped as soon as adverse reactions appear (positive test) or after a considerable amount has been consumed without reactions (negative test). An open food challenge (not double blind or placebo controlled) can be used to confirm negative results, in case of positive results a DBPCFC should be applied to exclude any bias [118]. In general a food challenge can be easier classified as positive the sooner symptoms appear and the more organ systems are affected. Moreover a study by Rolinck-Werninghaus [119] showed that elevated cow’s milk-specific IgE levels, young age and atopic dermatitis are factors that indicate a positive challenge outcome. Furthermore it is necessary to challenge the patient until clear objective symptoms occur without doing harm to the tested person by reaching the maximum response. Unfortunately DBPCFC are not suitable to estimate the risk of a reaction after CM consumption since augmentation factors cannot be excluded but it is useful to determine the minimum eliciting dose for an acute allergic reaction [120]. Despite the clear diagnosis this test has several disadvantages: it is very time consuming, costly, can only be performed under medical guidance and bears the risk of inducing severe anaphylactic reactions [115].

### 2.2 Skin prick test (SPT)

A SPT is a fast and inexpensive possibility to detect sensitization in IgE-mediated disorders. For this purpose a commercial CM extract or fresh milk or single allergen components and a saline-glycerine control are pricked with a lancet into the epidermis of a patient. If the patient has IgE antibodies against the food allergen, a wheal greater than the saline control will appear. The negative predictive value using fresh milk is excellent (>95%), unfortunately the specificity of this test is poor and it does not prove that the tested food component is the trigger [48]. Several studies tried to set up positive predictive values (PPV) but the results are conflicting: One study showed that a wheal diameter of 6 mm has a 95% predictive value of a clinical reaction in children with 2 years of age or younger and of 8 mm in children older than 2 years [121].

Vanto et al. found a 79% positive predictive value for wheal size ⩾3 mm when infants under 1 year were pricked with CM based formula [122]. Another research group set the 92% PPV at a wheal size ⩾8 mm [123] similar to Sporik for children older than 2 years [124]. Verstege et al. defined a 95% PPV at a wheal diameter of 12.5 mm in children with 22 months of median age [125] when pricked with fresh milk and Calvani et al. a wheal diameter of 15 mm in infants with mean age of 3.6 years [126]. In contrast, Costa et al. found that skin prick tests had only a 66.7% PPV in CM allergic children between one and five years with wheals ⩾3 mm and recommended the oral challenge as the best method for diagnosing CMA [127].

When comparing commercial extract with fresh milk in SPT, Calvani et al. [126] found that fresh milk shows the highest negative predictive value whereas casein shows the greatest positive predictive value. Another study presented the marked differences in protein composition among crude and commercially available allergen extracts used for SPT [128] leading to different test results. These differences and the fact, that skin test solutions may be contaminated, emphasize a need for additional forms of diagnostic tests e.g. *in vivo* tests using purified allergen components as single solutions or mixes [129].

### 2.3 Atopy patch test (APT)

APTs can be performed in patients with atopic dermatitis or gastrointestinal symptoms lacking specific IgE but also in patients with delayed reactions after CM consumption [130]. For this purpose allergens are applied, normally at the back of the patients for up to 48 h in a sealed patch and skin reactions are documented after the removal of the patches and after another 24–48 h. Furthermore this test is recommended for the diagnosis of eosinophilic esophagitis in adults and children and for the early diagnosis of gastrointestinal symptoms after CM consumption in preterm infants [52]. APT might be also beneficial in predicting oral tolerance in children with gastrointestinal symptoms suffering from non-IgE-mediated CMA [53]. Unfortunately reagents, application methods or guidelines for interpretation have not been standardized so far. For this reason several studies analysing the diagnostic value of APT still recommend the parallel use of multiple tests for the diagnosis of CMA [122,123,127,131–133].

### 2.4 Measurement of cow’s milk allergen-specific IgE

For this diagnostic test venous blood samples are obtained from patients. In the next step patients’ sera are exposed to solid matrix-bound allergens (skimmed CM) and then detected by a secondarily labelled antibody specific for the Fc portion of human IgE. Therefore the sensitivity of this kind of IgE determination is very high. Sometimes these tests deliver irrelevant positive results, making it necessary to always include the clinical history into the interpretation of the test results [48,134]. These IgE antibody assays are offered by Phadia (ImmunoCAP System), Siemens Healthcare Diagnostics (Immulite), Hycor Biomedical (HYTEC-288) and other companies [135].

The group of Sampson was one of the first research teams that described predictive values for IgE measurements. His group found that in 95% of patients with milk-specific IgE levels above 15 kUA/L clinical symptoms can be expected during an oral challenge [110,136]. Further studies from other groups reported that a 90% diagnostic value for milk allergy was 1.5 kUA/L (age 13–18 months), 6 kUA/L (age 19–24 months) and 14 kUA/L (age 25–36 months) [137,138]. In contrast one study group set a diagnostic value for 95% probability of allergy to CM at 46 kUA/L [139]. Van der Gugten described the 95% diagnostic value for IgE to CM at 7.5 kUA/L for infants below 2.5 years of age [140] and the group of Komata set the 95% probability for failing an oral challenge at 5.8 kUA/L (<1 year old), 38.6 kUA/L (13–24 months) and 57.3 kUA/L (>2 years old). The differences are mainly due to various study populations regarding selection criteria or age of participants or different criteria for determining a failed or passed challenge [141].

Although this diagnostic test has a high sensitivity, patients suffering from a non-IgE-mediated CMA cannot be captured with this analysis and have to be tested in a DBPCFC [48].

## 3 Improvement of diagnosis

### 3.1 Purified natural and recombinant cow’s milk allergens

The majority of diagnostic tests are based on natural allergen extracts lacking sufficient quality, such as absence of important allergens, the presence of contamination and undefined non-allergenic components leading to inaccurate diagnosis of CMA [142]. In the last years a lot of effort was put in the identification and characterization of relevant milk allergens.

Nowadays pure allergen molecules derived from natural allergen extracts or produced by recombinant expression allow precise diagnoses with identification of the disease-eliciting allergens [143]. Therefore messenger RNAs are isolated from bovine mammary glands and transcribed into cDNAs. These sequences that code for allergens are cloned into vectors and are expressed in different expression systems, mainly in *E. coli* [144] but also in insect cells if posttranslational modifications are necessary. With pure and well characterized allergens it is possible to map IgE, IgG and T cell epitopes using sera from CM allergic patients. The knowledge of the allergen structure, allergen characteristics and the position of the epitopes improves not only the investigation of the mechanisms underlying allergies but also the development of diagnostic tools. Moreover recombinant allergens can be produced in high quantities and endotoxin-free [145]. Another advantage is that allergens expressed in *E. coli* lack carbohydrates and therefore are not recognized by carbohydrate-specific IgE antibodies leading often to clinically irrelevant results. When recombinant allergens are used for diagnosis they have a defined quality and concentration and are composed of single isoforms whereas natural allergen preparations may be a mixture of different isoforms with various biological activities. Especially in case of the CM allergens αS1-casein and αS2-casein can be obtained separately by recombinant technology which is not possible by purification procedures from the CM extract. These pure allergens not only improve the diagnosis, they also facilitate an important progress from extract-based to component-resolved diagnosis (CRD). In addition, the use of recombinant purified proteins allows the identification of cross-reactive allergens and explains allergic symptoms after consumption of various foods [146,147]. However not all proteins expressed in *E. coli* have comparable characteristics as their natural counterparts or have a correct folding. Sometimes it is necessary to use eukaryotic expression systems to get correctly folded proteins.

The milk proteins α-lactalbumin, β-lactoglobulin, αS1-casein, αS2-casein, β-casein and κ-casein have been expressed in *E. coli* and were tested in several studies regarding their purity, fold and IgE reactivity [21,54,58,148,149]. In the future it will be possible to use single recombinant allergens or a mix of several recombinant cow’s milk allergens which contain the allergen repertoire and all relevant IgE epitopes but lack disturbing non-allergenic materials as already shown for tree and grass pollen allergy [150].

**Fig. 2:**
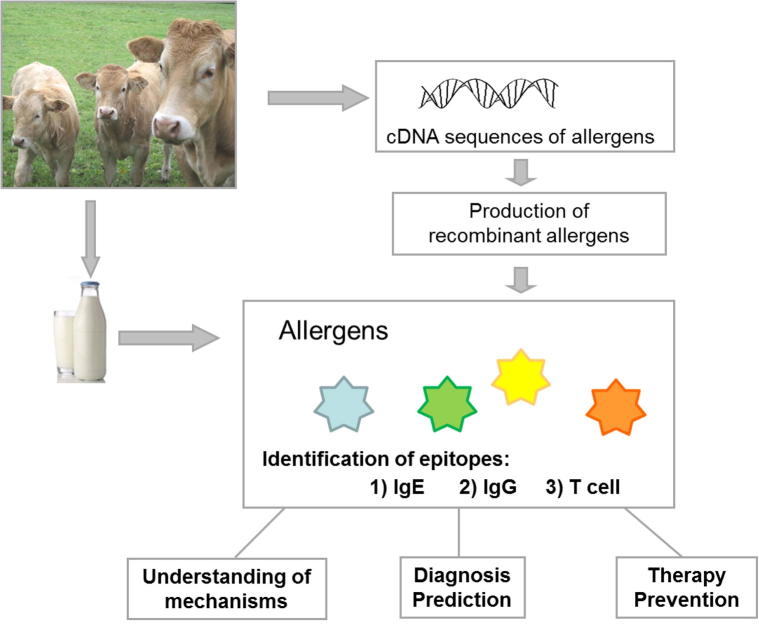
From DNA sequences to understanding the mechanisms of cow’s milk allergy which helps to improve diagnosis and allows designing strategies for therapy and prevention. Messenger RNA isolated from bovine mammary glands is converted into cDNA and then used for the production of recombinant allergens similar to natural allergens. This application can be used for the location of IgE, IgG and T cell epitopes in order to develop suitable treatments.

Furthermore this technological approach allows the design of strategies e.g. such as hypoallergenic derivatives for allergy therapy (Fig. 2).

### 3.2 Component-resolved diagnosis (CRD) and microarray technology

The serological test systems currently used in clinical praxis like ELISA, RAST or CAP-FEIA are not suitable for component-resolved diagnosis because they are designed as single allergy tests and would require a big amount of patients’ sera, are work- and time-intensive and expensive. In the last years, progress in the fields of molecular biology, biochemistry and biotechnology led to the development of protein microarray chips or other multiplex technologies [147,151,152]. Currently commercially available protein microarrays allow the detection of IgE reactivity to 103 allergenic molecules (ImmunoCAP ISAC-CRD 103, Phadia, Uppsala, Sweden) in routine use or to even more allergens in research settings.

In general, customized microarray platforms contain purified natural CM allergens [153] or recombinant allergens and peptides thereof. These components are spotted onto nitrocellulose membranes or activated glass surfaces in triplicates.

**Fig. 3:**
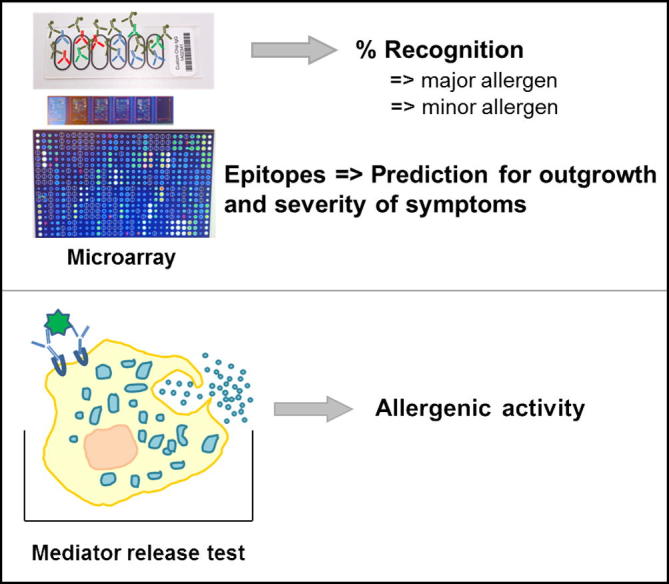
Use of recombinant allergens for diagnosis. Recombinant allergens can be spotted onto microarrays and tested with small samples of patients’ sera. Furthermore the allergenic activity can be measured with mediator release tests.

Protein and peptide microarrays are new diagnostic tools that allow measuring the IgE and IgG levels to CM allergens and classify them into major and minor allergens (Fig. 3) [154]. Especially with peptide microarrays it is possible to determine the diversity of IgE antibodies binding to sequential epitopes [155]. These tests help to study the development of allergic immune responses early in childhood and allow the monitoring of immune responses during the natural tolerance induction [144]. The knowledge of the IgE recognition sites facilitates the design of allergy vaccines. Due to the enormous potential of these tests, studies on microarrays are increasing [80,153,156–162]. In a recent study it was shown that the combination of microarrays with mediator release tests allows predicting the presence and the severity of clinical symptoms and furthermore provides interesting additional diagnostic information such as outgrowth [21]. Several other studies have confirmed that CRD with characterized and pure allergens is useful in predicting presence of allergy compared with whole extracts although CM-specific IgE is already a good prognostic marker for outgrowth [163]. Microarrays have a good ability to predict food challenge test results which might reduce the number of challenges in the future [159]. It has been shown by Beyer et al. that binding of IgE antibodies to distinct epitopes of CM allergens is a hint for a persistent CMA [164]. Further well-designed studies are necessary to correlate useful molecular diagnostics and biological markers with patients IgE recognition patterns and clinical symptoms [154]. On the one hand it is necessary to consider that competing IgG antibodies may influence IgE binding by competition with IgE for the allergen especially when small amounts of CM allergens are spotted [165]. This has to be kept in mind when changes of IgE levels are determined during the course of an immunotherapy, which induces high levels of IgG antibodies. On the other hand the interference of IgG antibodies with IgE is more similar to the *in vivo* situation e.g. skin prick testing.

The routine application of microarray technology will allow the determination of reactivity profiles of allergic individuals to large numbers of disease-causing allergens within a single assay that requires only minute amounts of patients’ sera which is of particular importance in case of diagnosis of milk allergy in infants and children. A good characterization of patients’ IgE reactivities including determination of cross-reactive allergens may also represent a first step towards the development of new immunotherapeutic strategies for CMA similar to those that are developed based on recombinant allergens for respiratory allergies [144,150,154,166]. Another application is to study the course of IgE reactivity profiles [167].

### 3.3 Basophil mediator release/basophil activation tests

Different methods are nowadays available to test if IgE antibodies are not only capable of binding allergens but also induce mediator release in order to avoid provocation tests [168]. In basophil histamine and leukotriene C_4_ release assays, basophils are incubated with different concentrations of allergens which crosslink FcεRI-bound IgE and induce mediator release [165] (Fig. 3). For this purpose, basophils from sensitized patients, IgE-depleted stripped basophils from healthy donors, basophil cell lines or animal cell lines transfected with human IgE high affinity receptors are incubated with patients’ sera containing IgE antibodies. Released mediators are measured by radioimmunoassay or enzyme-linked immunosorbent assay [169,170]. Another test method includes the measurement of allergen-induced basophil activation by flow cytometry by analysis of the basophil activation markers CD203c or CD63. These basophil tests help to determine the clinical course of CMA and to make the decision if food challenges should be performed [21,171–175].

A promising strategy for diagnosing CMA is the combination of two techniques: microarrays and basophil activation assays. As performed by Lin et al. microarrays were exposed to effector blood cells carrying IgE representing an *in vivo* assay very close to the mechanisms taking place during an allergic reaction [147,176]. Another approach that combined microarray technology with mediator release assay allowed the prediction of outgrowing the allergy [21].

In order to establish mediator release assays as diagnostic tests it is necessary to standardize the allergens/allergen extracts and also the procedure (concentration of cells and allergens, incubation times, cut off values, quality control reagents, methods) [165].

### 3.4 IgG/IgA antibodies

Measurement of cow’s milk-specific IgE antibodies has become a standardized test method, differently from non-IgE-mediated hypersensitive reactions, where *in vitro* cellular or antibody-based test systems are still controversially discussed [41,177,178]. The idea of testing IgG when IgE is lacking is based on observations from the early 1980s that IgG_4_ may induce mediator release from basophils [178]. Studies that tried to investigate IgG and IgA levels to CM proteins in allergic and healthy individuals gave controversial results [44,179–184]. In our study, IgG_1–4_ subclass and IgA antibody levels to purified recombinant αS1-casein, αS2-casein, β-casein, κ-casein, α-lactalbumin and β-lactoglobulin were determined in different patient groups by ELISA. It was not possible to distinguish CM protein intolerant patients from persons without CM protein intolerance [54] which is in agreement with the position paper from Stapel [178] that discourages from the diagnosis of food intolerance by IgG tests.

## 4 Therapy and prevention

### 4.1 Avoidance of cow’s milk and dietary treatment

The current treatment of CMA is the elimination of CM from the daily nutrition [185]. After a detailed clinical history is obtained and cow’s milk-specific *in vitro* tests identify the allergy-eliciting food components, CM needs to be excluded from the diet under medical care. Some patients suffering from CMA may tolerate small amounts of extensively heated or baked milk [186]. A recently published study showed that tolerance of baked milk products is a prognostic indicator for development of tolerance to CM and that inclusion of baked milk products into the daily diet has a positive influence on the development of tolerance [187]. It is necessary that doctors keep the improvement of symptoms, nutritional deficiencies, increase in cost and time in mind when an appropriate diet is selected [188]. Especially in case of CMA which mainly affects infants, parents are advised to administer milk formulas until at least 2 years of age. In general, extensively hydrolyzed formulas (eHF) are the most suitable alternative and are tolerated by 95% of milk allergic children [48,189,190]. Compared to amino acid formulas the eHF are cheaper and show similar clinical outcomes [191]. Only in case of persistence of symptoms an amino acid formula needs to be prescribed. It is important to administer a well-balanced diet with a proper calorie/protein ratio, amino acid composition and calcium source. Also the reintroduction after outgrowth of CMA should be done under medical guidance [13,48,116].

A recently published study has shown that the non-invasive milk APT test can be used for early diagnosis of CMA in preterm infants and helps to decide which type of formula (standard CM formula, extensively hydrolyzed CM formula, amino acid based formula) is administered [52].

Another important aspect that needs to be kept in mind is that milk from other mammals is often proposed as a suitable substitute for cow’s milk. However several case reports and studies have shown that there are high amino acid sequence homologies between the allergens from cow, sheep and goat [27]. Therefore milk from sheep or goat is not an appropriate alternative for the majority of CMA infants. Mare’s, camel’s and donkey’s milk differ from cow’s milk regarding their protein composition and are therefore better accepted [192,193]. There are only few reports that described the clinical reactivity and sensitivity to human milk, however the clinical relevance is still not known [37,194].

Patients with CMA should not include CM derived products such as cheese, yoghurt, butter and cream into their diet [60]. Apart from mammals’ milk also soy milk needs to be consumed with care. In up to 15% of CMA infants, soy milk induces symptoms due to its highly allergenic potential [2]. Similarly frequent, CMA patients suffer from beef allergy, due to BSA, that is present in both foods [195]. BSA is not only present in milk and beef, but also in cow’s dander resulting sometimes in allergic and respiratory symptoms after CMA patients have contact with cows [196].

### 4.2 Medical treatment

The treatment of CMA includes oral antihistamine for mild cutaneous or digestive reactions and an epinephrine auto-injector for systemic or respiratory reactions [2,48].

Other non-specific treatments include the use of monoclonal anti-IgE antibodies which help to reduce the free IgE antibodies in the blood of allergic patients. This leads to a reduction of basophil activation and an increased threshold dose [197]. Furthermore the administration of prebiotics which favour the colonization of the gastrointestinal tract was tested in CM allergic individuals. However its beneficial effect is still under debate [198,199] although some studies point to a decreased severity of atopic disease, reduced inflammation and faster induction of tolerance when probiotics are added to extensively hydrolyzed formulas [199–204]. Other non-allergen-specific treatments include the administration of Chinese herbs or cytokine therapy [205].

### 4.3 Immunotherapy (IT) and future strategies for specific immunotherapy

Although immunotherapeutic treatment has already a long history and is well established for respiratory allergies [142], attempts to reduce allergic symptoms in food allergic patients with subcutaneous immunotherapy were tested for peanut allergy but stopped after severe reactions [206]. So far there is no approved therapy on the market. Other possibilities could be different immunomodulatory treatments such as oral or sublingual immunotherapy [197,207] or safer injections using well defined recombinant allergens with reduced allergenicity [208]. Therefore studies comparing the long-term consequences and the effectiveness of the different immunotherapies in contrast to the risk benefit need to be performed [186].

**Fig. 4:**
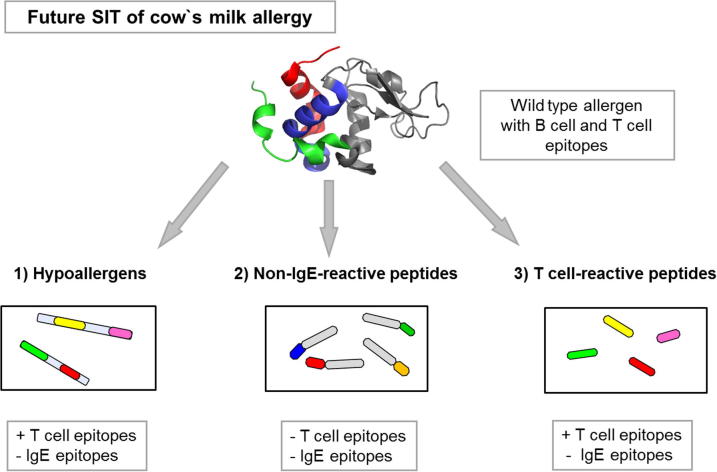
Allergen derivatives and peptides are important tools for the development of specific immunotherapy (SIT) of cow’s milk allergy. These hypoallergenic molecules contain T cell epitopes but lack IgE epitopes. Peptides without T cell epitopes, with no or reduced IgE binding activity can be linked to a carrier that stimulates carrier-specific T cell help. Furthermore T cell reactive peptides can be used for therapy and prevention.

One attempt for immunotherapy of CMA could be the development of a vaccine that contains recombinant hypoallergenic derivatives of the major CM allergens that have the majority of T cell epitopes but lack IgE epitopes (Fig. 4). Due to the immunogenicity of hypoallergens blocking IgG antibodies can be induced. The approach of site-directed mutagenesis was already applied for the major apple allergen Mal d 1, where 5 point mutations were performed. The artificially modified molecule showed a reduced IgE reactivity, allergenic activity in the basophil histamine release and lower allergenicity when tested in a DBPCFC. However care needs to be taken that the modification of the proteins does not result in the induction of new epitopes [209].

Similarly, the introduction of 2 mutations into the calcium-binding domain of the major fish allergen Cyp c 1 resulted in a molecule with reduced IgE reactivity and allergenic activity when tested in basophil histamine assays and skin prick tests [208,210]. This molecule is now tested in patients within the European research programme FAST [211].

Other approaches such as T cell peptides, peptide carrier fusion proteins and genetic vaccines were up to now only tested for respiratory allergens but not for food allergens. The idea behind the concept of T cell peptides is the application of T cell epitopes without IgE epitopes, which should be capable of stimulating allergen-specific T cells. The main principle of peptides coupled to carriers (i.e. rhinovirus-derived coat proteins VP1, PreS domain of hepatitis) is the reduction of immediate and late phase reactions during treatment, together with the stimulation of carrier-specific T cells, which provide help for the induction of blocking IgG antibodies [212–215] (Fig. 4). In addition, the production of regulatory T cells leading to permanent tolerance is also investigated [216].

### 4.4 Oral immunotherapy (OIT)

Oral immunotherapy has shown some promising improvements in life quality in patients with severe and persistent CMA [217,218]. However, it is not recommended for routine practice because controlled studies testing standardized protocols and outcome measurements are missing [219]. Several study groups have investigated the beneficial effect of OIT and found interesting results: In general increasing doses of CM are given in a special sequence: initial dose escalation during a controlled setting, then a regular consumption of tolerated doses during a build-up phase which is followed from a maintenance dose at home [197,220]. Animal studies have shown that high doses of antigen induce non-responsiveness resulting from anergy or deletion of antigen-specific T lymphocytes, whereas administration of continuous low doses induces regulatory T cells [220,221]. The success rate of the CM oral immunotherapy varied from 37% to 70% [222–224]. Longo et al. [223] showed that oral immunotherapy is an efficient treatment in CM allergic children with severe systemic reactions by inducing tolerance in 36% of 30 treated children. Furthermore it was possible to induce a higher threshold level of accepted CM (5–150 ml) in 54% of patients. In the study of Skripak et al. the induction of milk-specific IgG levels, predominantly IgG_4_, as a beneficial result of OIT could be shown. Although the threshold levels were increased in the treated group, the milk-specific IgE levels did not change significantly in the treatment or control group [222]. Narisety et al. showed similar results with increased tolerance to CM in the treated group but pointed out very clearly that adverse reactions were common and completely unpredictable [224]. Another attempt showed that oral desensitization in combination with omalizumab therapy could be induced more rapidly and without severe side effects in CM allergic patients [225]. A recently published study showed that a combination of milk together with interferon-γ increased the tolerance effect of oral immunotherapy by stimulation of allergen-specific IL-10-producing B cells [226].

Standardized protocols that include the optimal dose, degree of protection, ideal duration, safety, efficacy for different ages and severity of adverse reactions need to be designed [220,227]. It is known that the mechanisms that take place during immunotherapy are a decrease in milk-specific IgE, a decrease in basophil mediator release and an increase in blocking antibodies such as IgG_4_ and eventually induction of regulatory T-cells [185].

### 4.5 Sublingual immunotherapy (SLIT)

During this therapy milk is kept under the tongue in increasing dose in the rush period and continued for weeks to months during the maintenance period [228].

One study investigated the effect of SLIT with CM in a small cohort of patients (*n* = 8), where milk was kept under the tongue for 2 min. This treatment showed an increase in the threshold dose after 6 months [229]. Another recently published study by Keet et al. [230] compared the efficacy of SLIT and OIT and showed that OIT is more efficient for desensitization to CM probably due to the higher treatment dose which is in the range of several grams. However systemic side effects were also more common during OIT compared to SLIT. Upcoming studies need to address the selection of the optimal dose for SLIT in order to improve efficacy.

### 4.6 Epicutaneous patch (EPIT)

Within a small study it was shown that the treatment of CM allergic children with epicutaneous patches containing skimmed milk powder that were applied for 48 h each week for 3 months induced a higher milk tolerance level. However, side effects as pruritus and eczema appeared often and the immunological mechanisms underlying this treatment are unknown [231].

### 4.7 Cow’s milk allergy prevention

The guidelines for Europe and America recommend the exclusive breast feeding for 4–6 months and a delayed introduction of solid food components in infants with atopic risk [232]. However newer trials have shown that an early introduction of possible food allergens is beneficial and that those infants suffer less frequently from CMA. This would allow designing different prevention strategies in the future. One could be the administration of hydrolyzed formulas that contain tolerogenic peptides (Fig. 4) and testing if this induces tolerance which might be detectable as lack of allergen-specific humoral and cellular immune responses. Another option could be the feeding of infants with CM based formulas supplemented with prebiotics that has shown a beneficial effect in the reduction of atopic dermatitis [233].

## 5 Conclusions

The diagnosis of CMA has been improved in the last years due to the production of standardized testing materials such as purified natural allergens and recombinant proteins. With the use of defined and well characterized allergens it was possible to advance the extract-based into a component-resolved diagnosis which paved the way for a personalized diagnosis. So far avoidance of CM is the current treatment and oral immunotherapy is only performed in specialized settings. It will be one of the future goals to develop new forms of immunotherapy which reduce the risk of severe side effects and are more effective.

## Figures and Tables

**Table 1:** Main characteristics of cow’s milk allergens, adapted from Jost [62] and the IUIS allergen nomenclature (http://www.allergen.org) [21,59,61–66].

	Allergen name	Protein	Conc. (g/L)	Size (kDa)	No. of aa/molecule	pI	Prevalence (% of patients)	Microarray results (% of patients) [21]	Allergenic activity (% of patients) [21]
Whey (20%) (∼5 g/L)	Bos d 4	α-Lactalbumin	1–1.5	14.2	123	4.8	0–67	63	12
	Bos d 5	β-Lactoglobulin	3–4	18.3	162	5.3	13–62	50	19
	Bos d 6	Bovine serum albumin	0.1–0.4	66.3	582	4.9–5.1	0–76	4	1
	Bos d 7	Immunoglobulins	0.6–1.0	160			12–36		
		Lactoferrin	0.09	80	703	8.7	0–35	5	3
Whole casein (80%) (∼30 g/L)	Bos d 9	αS1-casein	12–15	23.6	199	4.9–5	65–100	49	26
	Bos d 10	αS2-casein	3–4	25.2	207	5.2–5.4			
	Bos d 11	β-Casein	9–11	24	209	5.1–5.4	35–44	44	35
	Bos d 12	κ-Casein	3–4	19	169	5.4–5.6	35–41	30	26
